# Deep Neuromuscular Blockade in Laparoscopic One-Anastomosis Gastric Bypass (OAGB): A Randomized Controlled Trial

**DOI:** 10.1007/s11695-026-08799-8

**Published:** 2026-06-16

**Authors:** Nikolas Drakos, Stella Antoniou, Ioannis D. Kostakis, Diamanto Aretha, Sofia Bregianni, Christina Kalogeropoulou, George Skroubis

**Affiliations:** 1 Department of Surgery, Medical School, University of Patras, Patras, Greece; 2 Department of Anesthesiology and Intensive Care, Medical School, University of Patras, Patras, Greece; 3 Department of Radiology, Medical School, University of Patras, Patras, Greece

## Abstract

**Introduction:**

Optimal visibility during Laparoscopic One-Anastomosis Gastric Bypass (OAGB) is challenging. This study compares moderate and deep neuromuscular blockade (NMB) for surgical field quality, respiratory and hemodynamic outcomes, atelectasis rates, pain, and bowel recovery.

**Methods:**

This randomized controlled trial involved adults with severe obesity (ASA II/III) undergoing OAGB with general anesthesia. Patients were assigned to deep or moderate NMB, monitored by acceleromyography. Surgical workspace quality was measured using the Leiden Surgical Rating Scale. Collected intraoperative data included workspace, operative time, pulmonary mechanics, hemodynamics, and oxygenation; postoperative outcomes covered pain scores, analgesic use, time to first flatus, complications, hospital stay, and atelectasis via chest CT.

**Results:**

Fifty-five patients were included (moderate: *n* = 28; deep: *n* = 27), with comparable baseline characteristics. NMB depth did not affect pulmonary mechanics. Deep NMB significantly improved intraoperative surgical field conditions (*p* < 0.001) and reduced operative time by approximately 10 min, with regression analysis confirming an 11.5 - minute reduction (*p* = 0.044). Hemodynamics and oxygenation were similar between groups. Patients in the deep NMB group reported lower postoperative pain scores, but comparable analgesic consumption. Length of hospital stay (*p* = 0.879), and incidence of atelectasis (*p* = 0.831) did not differ between groups. No association was found between BMI, NMB depth, and postoperative atelectasis.

**Conclusions:**

Deep NMB improves surgical conditions and shortens operative time for laparoscopic OAGB at standard pneumoperitoneum pressure. While it reduced postoperative pain, fixed intra-abdominal pressure and opioid-based anesthesia may have limited assessment of other outcomes. Further studies using multimodal strategies are needed to assess its impact on recovery.

**Supplementary Information:**

The online version contains supplementary material available at 10.1007/s11695-026-08799-8.

## Introduction

Bariatric surgery provides lasting weight loss and can improve or resolve conditions like type 2 diabetes, heart disease, and high blood pressure [1, 2]. Laparoscopic approach is fundamental key in enhanced recovery after surgery (ERAS) protocols and offer benefits over open surgery [3]. However, abdominal muscle contractions and increased intra-abdominal pressure can restrict the operative field and reduce visibility. This issue is especially significant in patients with obesity due to their thicker abdominal walls, making optimal surgical conditions essential [4, 5].

Obesity increases surgical risks because of impaired lung mechanics, higher complication rates, and longer recovery. General anesthesia and pneumoperitoneum worsen these effects. Deep neuromuscular blockade (NMB) may enhance laparoscopic conditions, though evidence in bariatric surgery is limited [6–11]. The European Society of Endoscopic Surgery guidelines recommend using the lowest intra-abdominal pressure (IAP) that allows adequate visibility; evidence suggests low-pressure pneumoperitoneum with deep NMB is feasible and safe [12].

Current studies indicate that deep ΝMΒ may improve surgical visibility and potentially decrease postoperative complications [13]. Earlier studies involving thoracic surgery used objective imaging to examine deep NMB’s impact on postoperative atelectasis. This study applied similar imaging methods to evaluate postoperative pulmonary atelectasis [14].

This study compares moderate and deep NMB in laparoscopic One-Anastomosis Gastric Bypass (OAGB) surgery, focusing on surgical field quality, lung mechanics and hemodynamic parameters during surgery, immediate respiratory complications (such as atelectasis), pain, nausea, and bowel function recovery.

## Methods

This randomized, controlled clinical trial, conducted from August 2024 to June 2025, was approved by hospital’s Ethical Committee and registered prior to patient enrollment. All participants provided written informed consent. Eligible participants were adults aged ≥ 18 years with ASA physical status II or III scheduled for elective laparoscopic one anastomosis gastric bypass (OAGB), with a biliopancreatic limb length of 200–250 cm, tailored according to preoperative BMI. OAGB is a popular, technically simpler bypass bariatric procedure with effective and long lasting results in weight loss and disease remission [15]. Patients were excluded from the study if they were under 18 years of age, classified as ASA IV, pregnant or postpartum, not medically eligible for general anesthesia, had contraindications to NMB or apnea, were anticipated to require awake intubation or a surgical airway, presented with neuromuscular disorders, or declined participation in the study.

Patients were randomly assigned by permuted-block randomization to either deep or moderate NMB group. Intraoperative evaluation of the surgical workspace quality was performed using the five-point Leiden Surgical Rating Scale (LSRS) [16].

Each case involved two anesthesiologists and two anesthesia nurses; all trained in obesity anesthesia and neuromuscular monitoring. Both groups used identical surgical and anesthetic protocols, differing only by NMB depth. One experienced surgeon performed all elective laparoscopic OAGB procedures. Pre-anesthesia evaluation ensured fasting and ERAS guideline compliance.

On entering the operating theatre, patients received appropriate monitoring, vascular access and radial artery cannulation. Neuromuscular function was assessed with acceleromyography (TOFscan^®^) via ulnar nerve electrodes. All patients were preoxygenated with 100% oxygen for at least five minutes. Anesthesia was induced with propofol, remifentanil, and rocuronium based on ideal body weight (IBW), while NMB was monitored every five minutes. The rocuronium induction dose was 1 mg/kg (IBW) for both group patients. Maintenance involved continuous infusions of propofol and remifentanil, maintaining bispectral index (BIS) between 35 and 45. Mechanical ventilation was administered using a 50/50 oxygen-air mixture in volume-controlled mode, with end-tidal CO₂ maintained between 30 and 35 mmHg. Obesity-specific techniques and individualized Positive End-Expiratory Pressure (PEEP) were employed. PEEP levels were adjusted intraoperatively based on respiratory mechanics, particularly dynamic compliance, to ensure optimal lung recruitment. If dynamic compliance fell below 30 mL·cmH₂O⁻¹, recruitment maneuvers were initiated and incremental increases in PEEP were implemented at the discretion of the attending anesthesiologist. The baseline PEEP for all patients was set at 10 cmH₂O.

NMB was achieved with intermittent 10 mg rocuronium boluses to maintain deep or moderate NMB levels. After anesthesia induction, patients were placed supine with legs apart. Pneumoperitoneum was set up at 14 mmHg using a Veress needle, followed by five 12-mm trocar insertions for the bariatric procedure. Patients were assigned to groups through randomization: Group A was administered rocuronium to achieve deep neuromuscular blockade (TOF = 0, PTC 1–2), whereas Group B received rocuronium only as necessary for moderate NMB (TOF 1–2), with saline employed as a placebo to maintain blinding. Once targets were reached, the surgeon assessed the surgical field every fifteen minutes. The surgeon was blinded to group allocation; however, due to the nature of neuromuscular blockade and its effects on diaphragmatic movement and surgical conditions, complete blinding may not have been fully maintained during the procedure.

Patients received multimodal analgesia with paracetamol, parecoxib, and fentanyl during surgery. Intraoperatively, patient positioning alternated between supine and reverse Trendelenburg for optimal exposure. NMB was reversed with sugammadex, followed by extubation when the train-of-four ratio exceeded 0.9.

Intraoperative data collection involved a thorough evaluation of surgical field conditions using the LSRS and documentation of operative time. Pulmonary mechanics, including peak inspiratory pressure (PIP), plateau pressure (Pplat), mean airway pressure (MAP), PEEP, airway resistance (Raw), dynamic compliance (Cdyn), and driving pressure (DP), were systematically recorded. Hemodynamic parameters, oxygen saturation (SpO₂), and BIS values were continuously monitored and documented.

After surgery, patients were monitored in Post Anesthesia Care Unit (PACU); those with pain scores above 4 on the Numeric Rating Scale (NRS) received 100 mg IV tramadol. Multimodal pain management continued with scheduled IV paracetamol and parecoxib, plus intramuscular pethidine as needed.

Postoperative monitoring included pain scores in PACU and at set times on days 0 (8pm) and 1 (2pm) (NRS), along with analgesic use, hemodynamics, and SpO₂. Outcomes recorded were time to first bowel flatus, and immediate incidence of postoperative lung atelectasis, assessed via chest CT within 6 h postoperatively. Patients remained in the post-anesthesia care unit (PACU) for about two hours before being transferred to the ward. Computed tomography (CT) imaging was typically performed soon after admission to the ward. Vitrea software was used to analyze CT images, calculating aerated and atelectatic lung volumes; atelectasis proportion was the atelectatic volume divided by total lung volume (see supplementary Fig. 1). Documented early complications included postoperative bleeding, anastomotic leak, and any postoperative respiratory complications. Hypoxemia was defined as PaO₂<8 kPa, PaO₂/FiO₂ ratio < 40 kPa, or SpO₂<90% requiring supplemental oxygen.

The primary outcome of this study was intraoperative surgical field quality, assessed using the LSRS. Secondary outcomes included operative time, intraoperative pulmonary and hemodynamic parameters, postoperative pain scores and analgesic use, time to return of bowel function, length of hospital stay, and the incidence and extent of postoperative atelectasis assessed by chest CT.

### Statistical Analysis

The relationship between different NMB techniques and surgical field quality or atelectatic lung volume or has not been previously studied in obese and superobese patients undergoing laparoscopic bariatric surgery.

Sample size calculation was based primarily on detecting a clinically meaningful difference in LSRS scores between groups. In addition, the study was powered to explore differences in atelectatic lung volume as a secondary outcome, recognizing that variability in this measure may limit the ability to detect smaller between-group differences. We aimed to detect an absolute difference of 15 ml in atelectatic lung volume or a 0.8 mean difference in LSRS scores between groups, with 80% power and a 0.05 significance level (t-test, effect size 0.8). This required 52 patients (26 per group); accounting for potential dropouts, we recruited 28 per group.

Group characteristics were summarized using descriptive statistics. Normality was assessed with the Shapiro-Wilk test. For quantitative variables, Student’s t-test, Welch t-test, or Mann-Whitney U test were used, and for qualitative variables, Chi-squared or Fisher’s exact test were applied as appropriate. Repeated measures were analyzed with adjusted general (or generalized) linear models. Spearman’s rank correlation coefficient evaluated associations between quantitative variables. Multivariable linear regression included variables with *p* < 0.1 in univariate analysis and those selected based on scientific rationale and collinearity checks; multicollinearity was assessed by VIF. No formal adjustment for multiple comparisons was performed. The analysis was structured around a single predefined primary outcome (LSRS), while all other endpoints were considered secondary and exploratory. Results for secondary outcomes should therefore be interpreted cautiously. All tests were two-tailed with significance at *p* < 0.05. Statistical analyses were conducted using SPSS version 29 and GraphPad Prism version 10.6.1.

## Results

### Included Cases – Baseline Characteristics

**Fig. 1:**
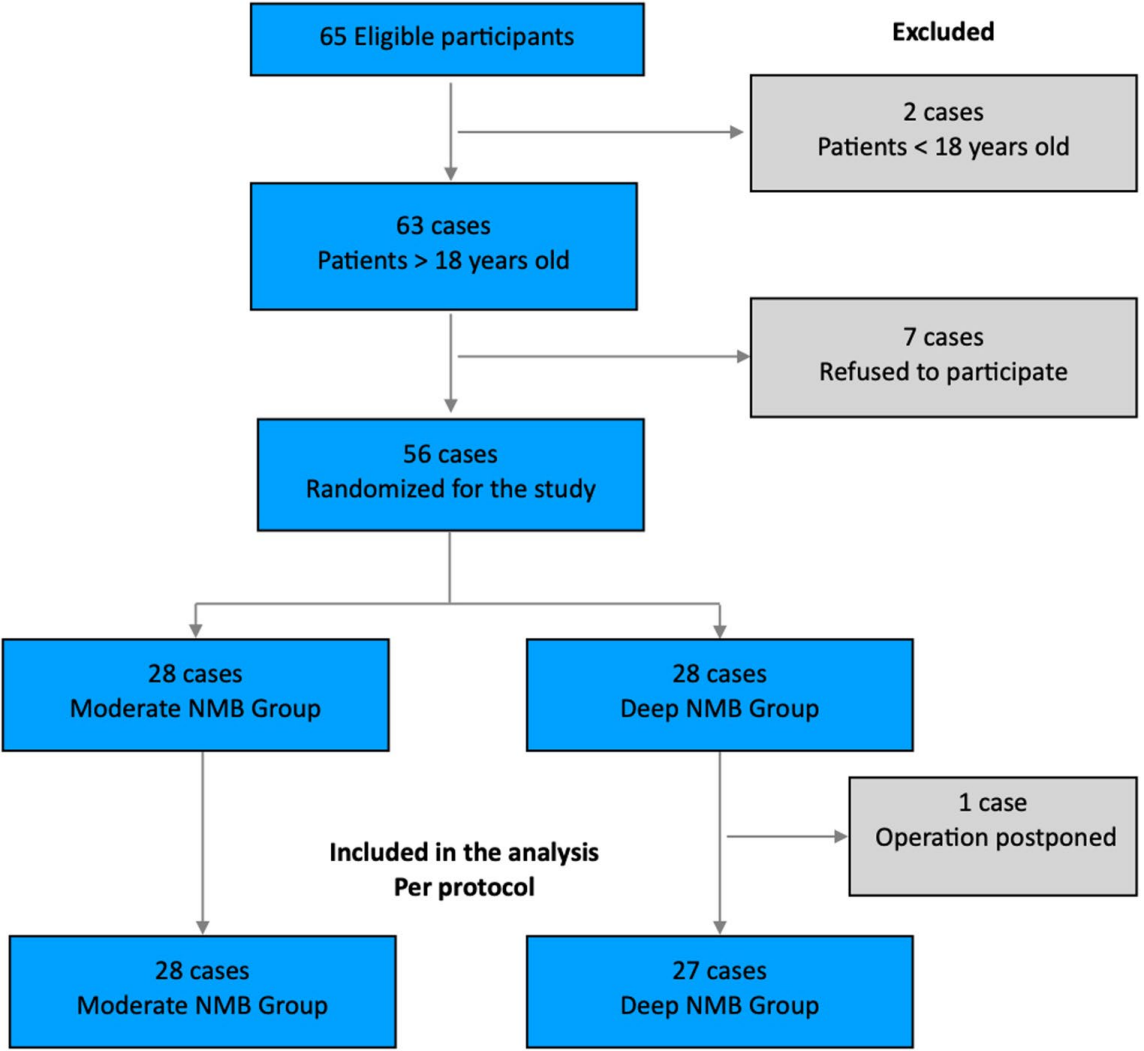
Study flowchart. NMB : neuromuscular blockade

Out of the 55 patients included in the study, 28 (50.9%) underwent moderate NMB, while 27 (49.1%) received deep NMB during surgery. The study flowchart is presented in Fig. 1. As detailed in Table 1, both groups were comparable across all baseline characteristics.

**Table 1:** Baseline Characteristics

Parameter	Moderate neuromuscular blockade (*N* = 28)	Deep Neuromuscular blockade (*N* = 27)	*P*-value
Gender	Male: 17 (60.7%) Female: 11 (39.3%)	Male: 11 (40.7%) Female: 16 (59.3%)	0.139
Age (years)	Mean (SD): 43.6 (10.3) Median (min-max): 44 (21–67)	Mean (SD): 43.3 (11) Median (min-max): 41.5 (23–61)	0.908
BMI	Mean (SD): 47.5 (6.6) Median (min-max): 46 (36.3–61.4)	Mean (SD): 49.4 (7.2) Median (min-max): 49.7 (38.7–64.3)	0.311
Height (cm)	Mean (SD): 174.8 (9.9) Median (min-max): 176 (153–193)	Mean (SD): 170.3 (9.1) Median (min-max): 170 (150–185)	0.091
Weight (kg)	Mean (SD): 145.7 (27.8) Median (min-max): 143 (103–210)	Mean (SD): 143.5 (25.7) Median (min-max): 140 (99–213)	0.766
Ideal body weight (kg)	Mean (SD): 67.6 (11.5) Median (min-max): 69.5 (46–87)	Mean (SD): 63.5 (9.9) Median (min-max): 61 (46–80)	0.082
Smoking	11 (39.3%)	12 (44.4%)	0.783

*BMI* Body mass index

### Intraoperative Parameters

### Ventilation Parameters

**Fig. 2:**
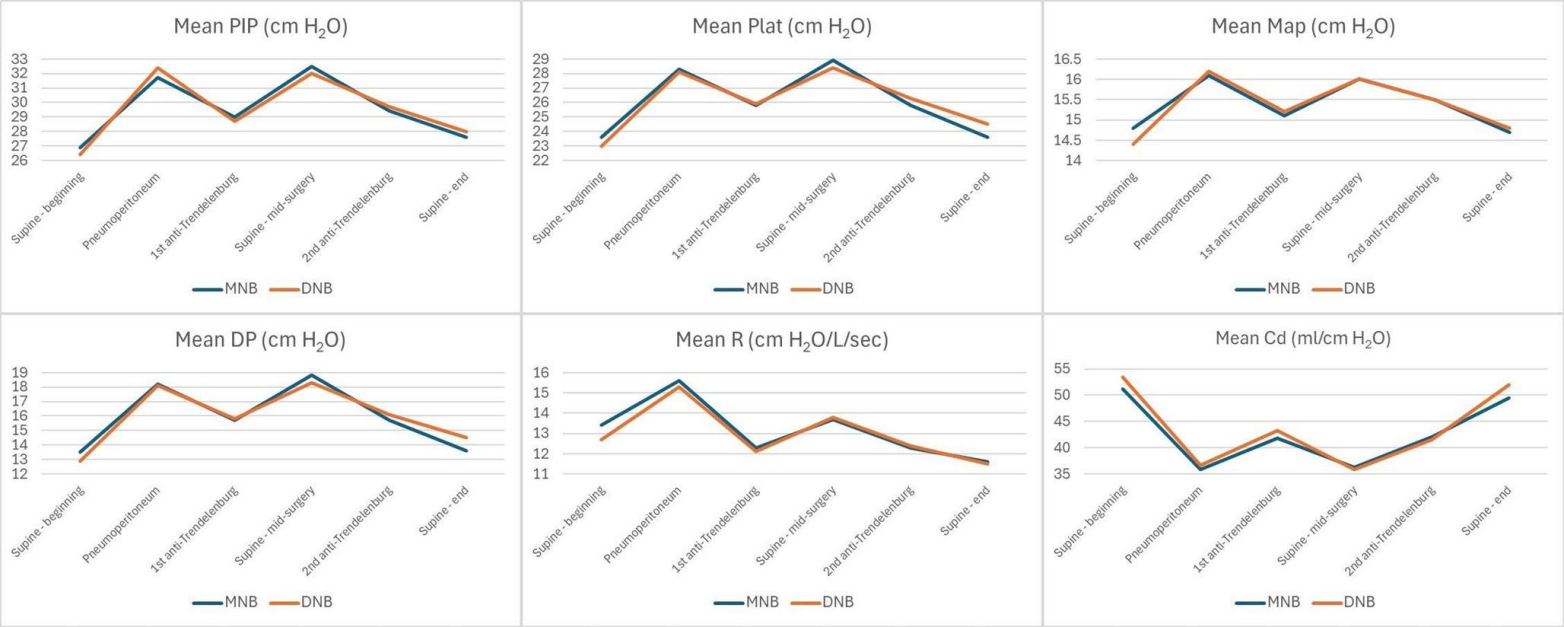
Ventilation parameters fluctuations throughout the operation period. MNB: moderate neuromuscular blockade, DNB: deep neuromuscular blockade, PIP: peak inspiratory pressure, Pplat: plateau pressure, Map: mean, DP: driving pressure, Raw: airway resistance, Cd: dynamic compliance

Ventilation parameters fluctuated significantly during the operation (*p* < 0.001; see Fig. 2). However, when analyzed using generalized linear mixed models, NMB depth showed no significant interaction with these fluctuations for PIP, Pplat, Map, DP, Raw, or Cd (all *p* > 0.8; see supplementary Table 1). No notable differences in ventilation parameters were found between moderate and deep NMB at any intraoperative timepoint (see Table 2).

**Table 2:** Ventilation Parameters at Different Operation Stages

Parameter	Supine – beginning	Pneumoperitoneum	1st anti-Trendelenburg	Supine – mid-surgery	2nd anti-Trendelenburg	Supine – end
PIP (cm H_2_O)	MNB Mean (SD): 26.9 (3.9) Median (IQR): 27 (6)	MNB Mean (SD): 31.7 (4.7) Median (IQR): 31 (7)	MNB Mean (SD): 29 (3.8) Median (IQR): 29 (5)	MNB Mean (SD): 32.5 (17) Median (IQR): 32.5 (6)	MNB Mean (SD): 29.4 (3.7) Median (IQR): 29 (6)	MNB Mean (SD): 27.6 (3.9) Median (IQR): 28 (6)
	DNB Mean (SD): 26.4 (4.8) Median (IQR): 26 (7)	DNB Mean (SD): 32.4 (4.8) Median (IQR): 33 (5)	DNB Mean (SD): 28.7 (3.8) Median (IQR): 29 (7)	DNB Mean (SD): 32 (3.9) Median (IQR): 33 (5)	DNB Mean (SD): 29.7 (3.7) Median (IQR): 30 (4)	DNB Mean (SD): 28 (4.6) Median (IQR): 27 (8)
	P-value: 0.636	P-value: 0.571	P-value: 0.743	P-value: 0.693	P-value: 0.782	P-value: 0.688
Pplat (cm H_2_O)	MNB Mean (SD): 23.6 (3) Median (IQR): 23 (5)	MNB Mean (SD): 28.3 (4.6) Median (IQR): 28 (7)	MNB Mean (SD): 25.8 (3.5) Median (IQR): 25.5 (4)	MNB Mean (SD): 28.9 (3.9) Median (IQR): 29 (6)	MNB Mean (SD): 25.8 (3.2) Median (IQR): 26 (4)	MNB Mean (SD): 23.6 (3.2) Median (IQR): 23 (4)
	DNB Mean (SD): 23 (3.8) Median (IQR): 22 (6)	DNB Mean (SD): 28.1 (4.5) Median (IQR): 27 (8)	DNB Mean (SD): 25.9 (3.2) Median (IQR): 26 (5)	DNB Mean (SD): 28.4 (3.4) Median (IQR): 29 (5)	DNB Mean (SD): 26.3 (3.1) Median (IQR): 26.5 (4)	DNB Mean (SD): 24.5 (3.5) Median (IQR): 24.5 (6)
	P-value: 0.536	P-value: 0.887	P-value: 0.917	P-value: 0.6	P-value: 0.6	P-value: 0.362
Map (cm H_2_O)	MNB Mean (SD): 14.8 (1.6) Median (IQR): 15 (2)	MNB Mean (SD): 16.1 (1.9) Median (IQR): 15.5 (2)	MNB Mean (SD): 15.1 (1.6) Median (IQR): 15 (2)	MNB Mean (SD): 16 (1.7) Median (IQR): 16 (3)	MNB Mean (SD): 15.5 (1.6) Median (IQR): 15 (2)	MNB Mean (SD): 14.7 (1.9) Median (IQR): 15 (3)
	DNB Mean (SD): 14.4 (1.8) Median (IQR): 14 (1)	DNB Mean (SD): 16.2 (1.7) Median (IQR): 16 (2)	DNB Mean (SD): 15.2 (1.4) Median (IQR): 15 (1)	DNB Mean (SD): 16 (1.5) Median (IQR): 16 (2)	DNB Mean (SD): 15.5 (1.2) Median (IQR): 16 (1)	DNB Mean (SD): 14.8 (1.7) Median (IQR): 15 (2)
	P-value: 0.534	P-value: 0.618	P-value: 0.942	P-value: 0.724	P-value: 0.659	P-value: 0.92
DP (cm H_2_O)	MNB Mean (SD): 13.5 (2.6) Median (IQR): 13.5 (5)	MNB Mean (SD): 18.2 (4.1) Median (IQR): 17.5 (8)	MNB Mean (SD): 15.7 (2.9) Median (IQR): 15 (3)	MNB Mean (SD): 18.8 (3.5) Median (IQR): 19 (5)	MNB Mean (SD): 15.7 (2.8) Median (IQR): 16 (3)	MNB Mean (SD): 13.6 (2.6) Median (IQR): 13 (3)
	DNB Mean (SD): 12.9 (3.6) Median (IQR): 12 (6)	DNB Mean (SD): 18.1 (4.4) Median (IQR): 17 (7)	DNB Mean (SD): 15.8 (3) Median (IQR): 16 (4)	DNB Mean (SD): 18.3 (3.3) Median (IQR): 19 (5)	DNB Mean (SD): 16.1 (2.9) Median (IQR): 16 (5)	DNB Mean (SD): 14.5 (3.6) Median (IQR): 14.5 (6)
	P-value: 0.476	P-value: 0.903	P-value: 0.682	P-value: 0.597	P-value: 0.599	P-value: 0.313
Raw (cm H_2_O/L/sec)	MNB Mean (SD): 13.4 (2.8) Median (IQR): 13 (5)	MNB Mean (SD): 15.6 (3.7) Median (IQR): 15 (6)	MNB Mean (SD): 12.3 (1.7) Median (IQR): 12 (3)	MNB Mean (SD): 13.7 (2.1) Median (IQR): 13.5 (3)	MNB Mean (SD): 12.3 (1.4) Median (IQR): 12 (2)	MNB Mean (SD): 11.6 (1.8) Median (IQR): 11 (3)
	DNB Mean (SD): 12.7 (4.3) Median (IQR): 12 (5)	DNB Mean (SD): 15.3 (5) Median (IQR): 14 (6)	DNB Mean (SD): 12.1 (2.9) Median (IQR): 11 (3)	DNB Mean (SD): 13.8 (3.3) Median (IQR): 13 (5)	DNB Mean (SD): 12.4 (2.5) Median (IQR): 12 (3)	DNB Mean (SD): 11.5 (2.7) Median (IQR): 11 (2)
	P-value: 0.121	P-value: 0.432	P-value: 0.229	P-value: 0.647	P-value: 0.426	P-value: 0.473
Cd (ml/cm H_2_O)	MNB Mean (SD): 51.2 (11.1) Median (IQR): 50.6 (16.9)	MNB Mean (SD): 35.8 (7.1) Median (IQR): 34.3 (7.3)	MNB Mean (SD): 41.8 (8.3) Median (IQR): 40.6 (8.4)	MNB Mean (SD): 36.2 (6.3) Median (IQR): 37.4 (9.6)	MNB Mean (SD): 42.1 (8.3) Median (IQR): 42.8 (9.4)	MNB Mean (SD): 49.5 (9.5) Median (IQR): 48.2 (12.6)
	DNB Mean (SD): 53.4 (16.8) Median (IQR): 52.7 (17)	DNB Mean (SD): 36.6 (9) Median (IQR): 36.9 (13.2)	DNB Mean (SD): 43.3 (10.3) Median (IQR): 40.4 (14.2)	DNB Mean (SD): 35.9 (8.1) Median (IQR): 37 (9.1)	DNB Mean (SD): 41.5 (11.1) Median (IQR): 40.5 (14)	DNB Mean (SD): 52 (17.1) Median (IQR): 49 (27.1)
	P-value: 0.993	P-value: 0.692	P-value: 0.69	P-value: 0.678	P-value: 0.812	P-value: 0.538

*MNB* Moderate neuromuscular blockade, *DNB* Deep neuromuscular blockade, *PIP* Peak inspiratory pressure, *Pplat* Plateau pressure, *Map* Mean, *DP* Driving pressure, *Raw* Airway resistance, *Cd* Dynamic compliance

#### Operating Field

The average LSRS score was higher with deep NMB than moderate NMB [moderate: median (IQR): 3.8 (0.5); deep: 4.7 (0.6); *p* < 0.001]. Multivariable linear regression, adjusted for gender, age, and BMI, confirmed this association (B: 0.737; 95% CI: 0.443–1.031; *p* < 0.001). See supplementary Tables 2 and 3 for details.

#### Operating Time

Operations with deep NMB averaged 10 min shorter than those with moderate blockade [moderate: median (IQR) 65 (29), deep: 55 (20), *p* = 0.007]. Multivariable regression showed deep blockade reduced operating time by 11.5 min (95% CI: -22.7 to -0.3, *p* = 0.044), adjusting for gender, age, and BMI. See supplementary Tables 2 and 4 for details.

#### Intraoperative Vital Signs

No significant intraoperative differences were found in the average vital signs between groups: SpO2 [moderate: median (IQR): 97.9% (1.5%), deep: median (IQR): 97.9% (1.7%), *p* = 0.705], heart rate [moderate: median (IQR): 72 (10), deep: median (IQR): 77 (11), *p* = 0.095], SBP [moderate: median (IQR): 125 (16), deep: median (IQR): 125 (14), *p* = 0.907], DBP [moderate: median (IQR): 73 (11), deep: median (IQR): 71 (8), *p* = 0.35], or MAP [moderate: median (IQR): 90 (12), deep: median (IQR): 89 (9), *p* = 0.539]. See supplementary Table 2 for details.

### Postoperative Outcomes

#### Postoperative Vital Signs

No statistically significant differences were found in the average vital signs during the first postoperative hour between moderate and deep NMB cases. SpO2, heart rate, SBP, DBP, and MAP values showed similar results for both groups (all p-values > 0.16). Further information is available in supplementary Table 5.

#### Postoperative Pain

Deep NMB led to lower postoperative pain scores than moderate NMB at all measured intervals (see supplementary Table 5). Adjusted analyses confirmed these findings. No significant differences were found in postoperative analgesia (parecoxib or pethidine use) (see supplementary Tables 6–8).

#### Passing Flatus and Duration of Hospital Stay

Regarding the rest of the studied postoperative outcomes, there was a slight advantage of deep over moderate NMB in terms of time to passing flatus [moderate: median (IQR): 1.5 days (1), deep: median (IQR): 1 (0), *p* = 0.044], whereas the duration of hospital stay was the same in the two groups [moderate: median (IQR): 3 days (0), deep: median (IQR): 3 days (0), *p* = 0.879]. Additional data are available in supplementary Table 5.

### Postoperative CT Chest Findings

**Fig. 3:**
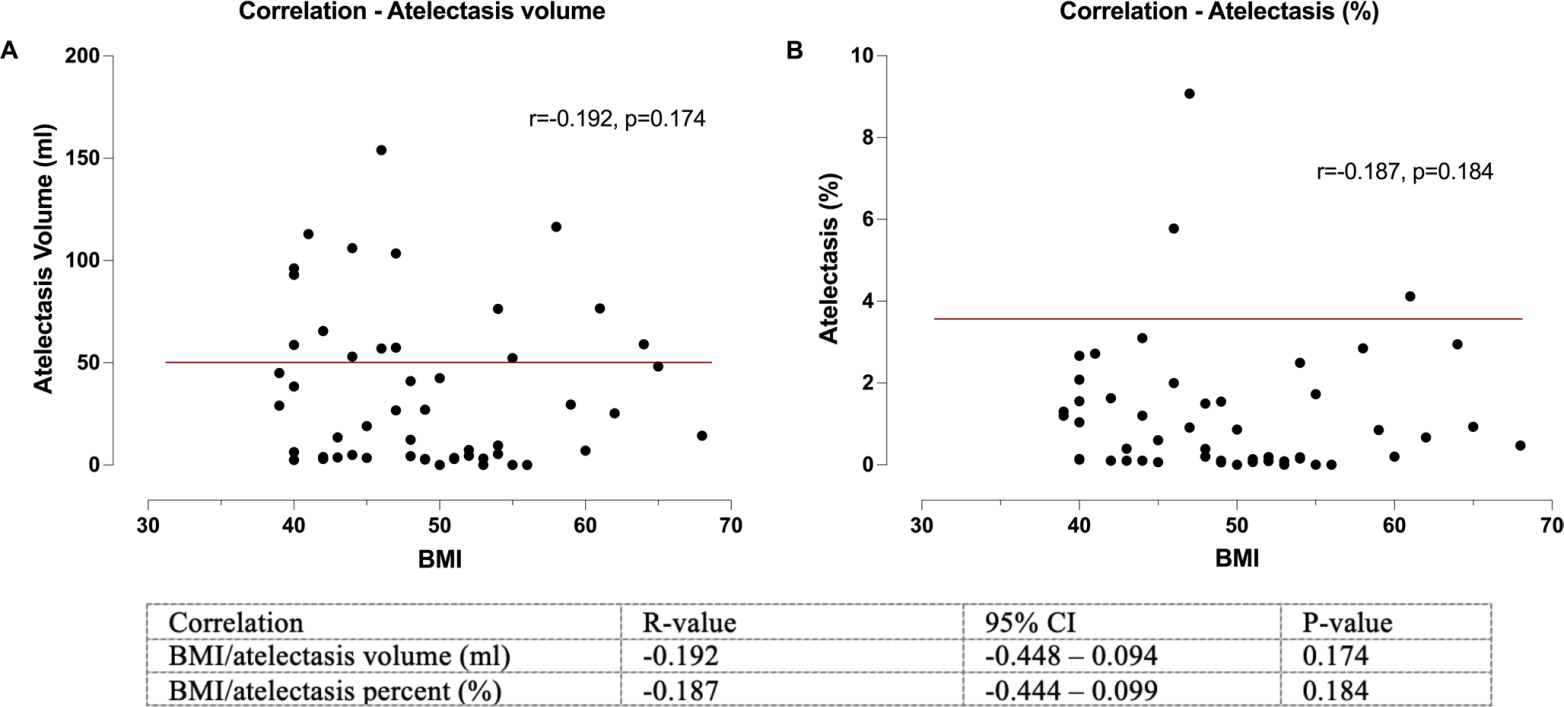
Association between Body Mass Index (BMI) and atelectasis volume (**A**), as well as atelectasis percentage (**B**), in relation to total lung volume

**Fig. 4:**
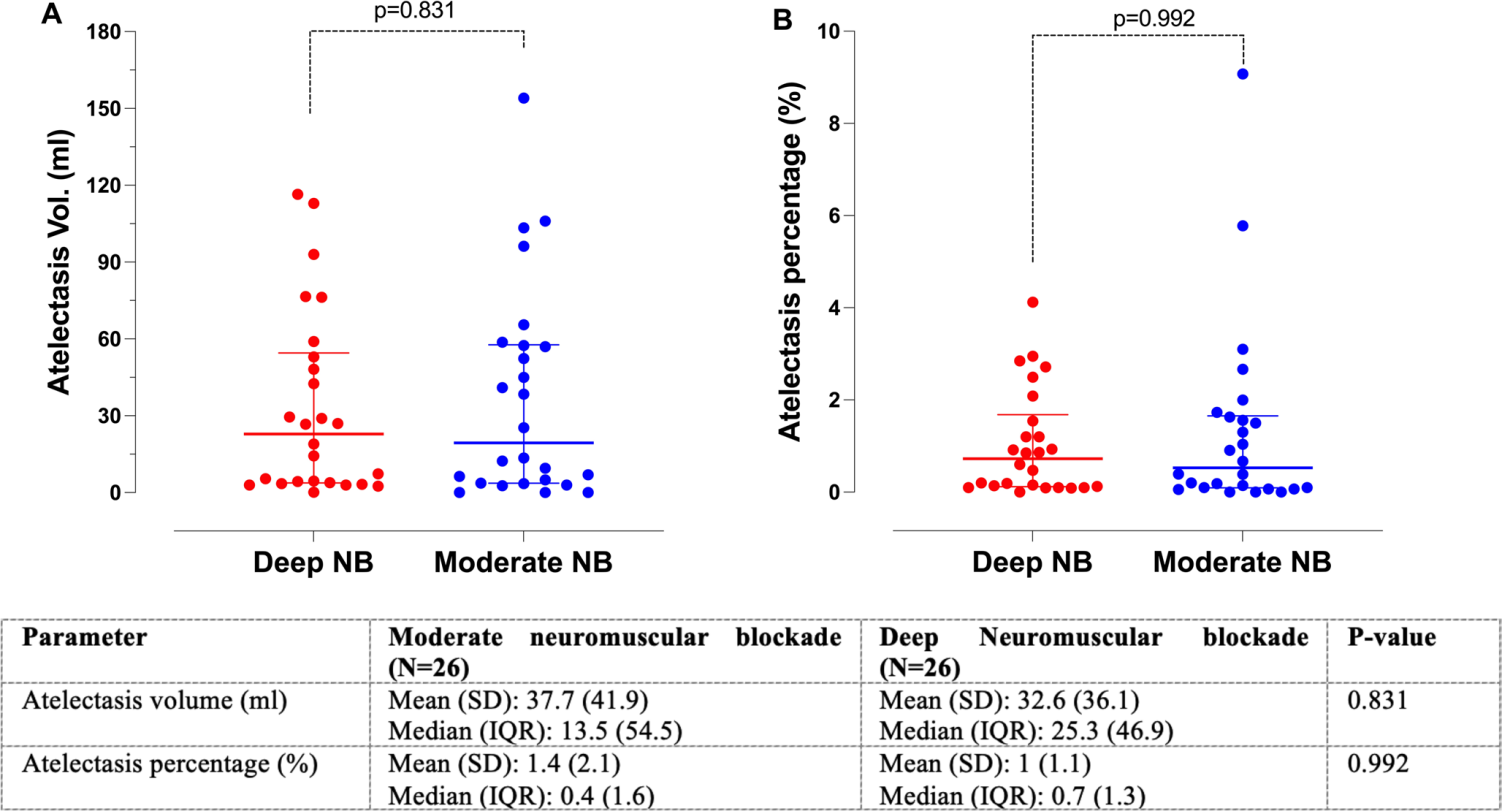
Comparison of atelectasis volume (**A**) and percentage (**B**) relative to total lung volume between the Deep and Moderate neuromuscular blockade (NMB) groups

BMI was not associated with either the volume (*r*=-0.192, 95% CI: -0.448 to 0.094, *p* = 0.174) or the percentage of lung atelectasis (*r*=-0.187, 95% CI: -0.444 to 0.099, *p* = 0.184) observed on postoperative chest CT (Fig. 3). Additionally, the depth of NMB did not influence atelectasis volume [moderate: median (IQR): 13.5 ml (54.5); deep: median (IQR): 25.3 ml (46.9), *p* = 0.831] nor its proportion relative to total lung volume [moderate: median (IQR): 0.39% (1.6%); deep: median (IQR): 0.67% (1.28%), *p* = 0.992] (Fig. 4). Multivariable linear regression analysis, after controlling for gender, age, BMI, smoking status, and operating time, confirmed these findings, indicating that deep NMB was not a risk factor for increased atelectasis, measured as absolute volume or percentage of total lung (refer to supplementary Tables 9 and 10).

## Discussion

This study assessed the effects of NMB depth on intraoperative respiratory mechanics, surgical field conditions, perioperative physiological stability, and postoperative incidence of lung atelectasis in obese patients undergoing laparoscopic bariatric surgery under general anesthesia. Deep NMB significantly improves surgical field conditions over moderate blockade, leading to shorter operating time by about 10 min. This reduction is likely due to increased precision, fewer interruptions, and better ergonomics for surgeons. Previous studies found no significant difference in operating time [9, 17, 18], possibly reflecting differences in study design, surgical complexity, or surgeon experience.

Our results align with a recent meta-analysis of 11 randomized controlled trials, which found that deep NMB leads to better surgical field conditions than moderate NMB [19]. Subgroup analyses of that meta-analysis showed inconsistent results, largely depending on whether surgical conditions were assessed once or multiple times during surgery. In bariatric surgery, single assessments found no difference between deep and moderate NMB, while repeated assessments revealed a significant advantage for deep NMB [13]. This variability highlights the importance of assessment methodology. In the present study, surgical field conditions were evaluated at 10-minute intervals during pneumoperitoneum, allowing for a more detailed assessment. The novelty of the present work does not lie in demonstrating this effect per se, but rather in evaluating its clinical implications within a specific perioperative context. In our study, both groups were managed under fixed pneumoperitoneum pressure, opioid-based total intravenous anesthesia, and controlled ventilation, conditions that may attenuate differences in patient-centered outcomes. Within this framework, our findings suggest that improved surgical workspace does not necessarily translate into measurable differences in postoperative recovery.

In the past, using deep NMB has led to worries about elongated recovery times and respiratory issues postoperatively. These concerns are mostly due to incomplete reversal and lasting NMB, which can impact the function of the upper airway and respiratory muscles [20]. Sugammadex has greatly improved safety by allowing rapid and reliable reversal of deep NMB caused by rocuronium or vecuronium [21, 22].

Our findings confirm that deep NMB is safe during the perioperative period. There were no significant differences in vital signs between groups, indicating that deep NMB does not compromise hemodynamic stability or oxygenation. These results are consistent with the findings of Torensma B et al., who similarly reported no statistically significant differences between deep and moderate neuromuscular blockade in patients undergoing bariatric surgery [18].

A key advantage of this research is the postoperative use of chest CT scans to objectively measure lung expansion during ventilation. Consistent with earlier studies of thoracic operations, deep NMB, when paired with appropriate reversal and lung-protective ventilation, did not influence the development of postoperative atelectasis [14]. Although CT-based evaluations of atelectasis have been reported in thoracic surgery, comparable imaging-based evidence in laparoscopic surgery comparing deep versus moderate NMB is lacking, with existing studies primarily focusing on surrogate outcomes such as surgical conditions, respiratory mechanics, and postoperative pain rather than direct radiological assessment [11, 17, 18, 23].

This study is the first to directly study the association between muscle blockade depth and postoperative atelectasis in laparoscopic surgery, with a specific focus on bariatric patients. BMI and NMB depth showed no correlation with postoperative atelectasis volume or percentage. Deep NMB was not a risk factor for increased lung atelectasis, even after adjusting for smoking and operation duration. Both groups had minimal atelectasis, likely due to effective intraoperative mechanical ventilatory management. Another key result from this study was that deep NMB was associated with lower postoperative pain scores; however, this finding should be interpreted cautiously given the study design. Despite statistically significant differences in pain scores, no corresponding reduction in postoperative analgesic consumption was observed. This apparent discrepancy may be explained by several factors. First, analgesic administration followed a protocol-driven approach based on predefined pain thresholds, which may have limited the sensitivity of this outcome to detect between-group differences. Second, the use of remifentanil-based anesthesia in both groups may have attenuated differences in postoperative analgesic requirements. Third, the magnitude of the observed difference in pain scores—approximately 1 point on the NRS scale—may be of limited clinical significance and below commonly accepted thresholds for a meaningful change in pain perception. Taken together, these findings suggest that while deep NMB may have a modest effect on postoperative pain perception, the clinical relevance of this effect remains uncertain. Nevertheless, even small reductions in pain scores may reflect improved surgical conditions and reduced tissue manipulation, although this hypothesis requires further investigation. While some earlier studies have found similar results, the data overall are inconsistent, with some reports finding clear benefits and others finding no significant differences [17, 18]. Our results are comparable to those reported by Toresman et al., who also observed lower postoperative pain with deep NMB [18]. On the contrary, a more recent study by da Silva MNC et al. found no significant differences in pain scores between NMB depths [17]. These discrepancies may be attributable, in part, to variations in pain assessment methodology. The present study focused on repeated assessments in the early postoperative period, when differences between the two levels of NMB are more profound. Although the mechanism is not fully understood, improved surgical conditions may reduce excessive trocar manipulation, abdominal wall injury and inflammation [24]. Deep NMB reduced the time to first flatus slightly when compared to moderate NMB, but the difference was not clinically significant. The depth of blockade had no effect on hospital stay or overall postoperative recovery.

The present study has several limitations. An important consideration in interpreting our findings is the use of a fixed pneumoperitoneum pressure of 14 mmHg in both study groups. While this approach ensured procedural standardization, it may have limited our ability to detect differences in postoperative outcomes. Under conditions of relatively high intra-abdominal pressure, the physiological and mechanical burden of pneumoperitoneum is likely to be similar between groups, potentially attenuating any effects of neuromuscular blockade depth on outcomes such as pain, pulmonary function, and recovery. It is plausible that the benefits of deep NMB on patient-centered outcomes may become more evident when combined with lower-pressure pneumoperitoneum strategies.

The use of 14 mmHg pneumoperitoneum in this trial should be interpreted as a standardized study condition and not as a recommended target pressure for contemporary laparoscopic or robotic bariatric surgery. Current practice should aim to use the lowest intra-abdominal pressure that provides adequate surgical exposure, particularly when deep neuromuscular blockade and stable insufflation technology are available. Because insufflated intra-abdominal volume or objective workspace volume at lower pressures was not measured, this study cannot determine whether similar surgical conditions could have been achieved with lower-pressure pneumoperitoneum.

Another important consideration is the anesthetic technique employed in this study. All patients received propofol–remifentanil total intravenous anesthesia with controlled mechanical ventilation and a strategy of relative hyperventilation. This approach is known to suppress diaphragmatic activity, respiratory drive, and abdominal muscle tone. As a result, adequate surgical conditions may be achieved even with moderate neuromuscular blockade, potentially attenuating the observable differences between moderate and deep blockade. Under more physiological ventilatory conditions, or in the setting of low-opioid or opioid-free anesthesia techniques, the contribution of neuromuscular blockade depth to surgical workspace may be more pronounced. Therefore, the present findings should be interpreted within the context of this specific anesthetic protocol, and their generalizability to other anesthetic strategies may be limited. Nevertheless, the use of a standardized anesthetic protocol across groups ensured internal validity and minimized confounding effects when comparing neuromuscular blockade depth.

In addition, no difference was observed in postoperative analgesic use, although this finding should be interpreted in the context of the opioid-based anesthetic protocol. The use of remifentanil-based total intravenous anesthesia may have influenced postoperative pain perception and analgesic requirements in both groups. Potent intraoperative opioid administration is known to suppress nociceptive responses and may mitigate differences in postoperative analgesic needs unless the degree of surgical trauma itself is substantially altered. Under these conditions, the absence of differences in postoperative analgesic consumption and recovery parameters between groups is not unexpected and should be interpreted with caution. These findings suggest that evaluating neuromuscular depth in isolation may be insufficient to detect its full clinical impact. Future studies should explore deep neuromuscular blockade as part of a multimodal perioperative strategy, including lower pneumoperitoneum pressures and opioid-sparing or opioid-free anesthetic techniques, where its potential benefits on patient-centered outcomes may be more apparent.

Furthermore, although the surgeon was formally blinded to group allocation, complete blinding may not have been achievable in practice due to the observable intraoperative effects of different NMB depths, such as variations in diaphragmatic movement and muscle relaxation. In addition, no formal assessment of blinding success was performed. This may have introduced bias in subjective outcomes, particularly the assessment of surgical field conditions using the LSRS. However, surgical field assessments were performed repeatedly during the procedure using a standardized scale, which may have mitigated some of this potential bias.

The timing of postoperative chest CT imaging represents an additional limitation. Although scans were performed within the first 6 h after surgery, the exact timing varied between patients due to logistical and clinical factors. Atelectasis is a dynamic phenomenon that may change rapidly in the immediate postoperative period, particularly in the context of ERAS protocols promoting early mobilization and respiratory exercises. Consequently, variability in CT timing may have introduced measurement variability and reduced the ability to detect subtle differences between groups. However, as this variability was not systematically related to group allocation, it is likely to have attenuated true differences rather than introduce bias.

Another limitation is the absence of objective measurements of abdominal working space. Although surgical conditions were assessed using the validated LSRS, no quantitative indices—such as insufflation volume, intra-abdominal workspace measurements, or compliance-based metrics—were recorded. The inclusion of such objective parameters could have strengthened the mechanistic interpretation of the observed improvement in surgical conditions with deep neuromuscular blockade. Future studies integrating both subjective and objective assessments of surgical workspace are warranted.

Finally, the small sample size may have limited the ability to detect clinically important differences, especially in secondary outcomes, and residual confounding cannot be excluded despite multivariable analyses. All patients had undergone one-anastomosis gastric bypass, so results may not generalize to other bariatric or non-bariatric surgeries. Additionally, the tightly controlled clinical conditions, including specific ventilation protocols and use of sugammadex, may not reflect typical practice, limiting external validity.

These findings highlight that the clinical impact of deep NMB may be highly context-dependent. In particular, when pneumoperitoneum pressure is not reduced and opioid-based anesthesia is used, improvements in surgical conditions may not translate into downstream benefits for the patient. This underscores the importance of evaluating NMB depth as part of an integrated perioperative strategy.

## Conclusions

Deep NMB improves surgical field conditions and is associated with shorter operative time in Laparoscopic OAGB surgery performed under standard pneumoperitoneum pressure. While lower postoperative pain scores were observed, the study design—particularly the use of a fixed intra-abdominal pressure and opioid-based anesthesia—may limit the ability to detect differences in other patient-centered outcomes. Further studies incorporating a multimodal perioperative strategy, including lower pneumoperitoneum pressures and opioid-sparing or opioid-free anesthetic techniques are needed to better evaluate the potential impact of deep neuromuscular blockade on postoperative recovery.

## Supplementary Information

Supplementary Material 1

Supplementary Material 2

Supplementary Material 3

## Data Availability

The authors confirm that the data supporting the results and conclusions of this study are available within the article. Individual participant and additional data are available on demand.

## Abbreviations

OAGB: One Anastomosis Gastric Bypass
NMB: Neuromuscular Blockade
ERAS: Enhanced Recovery After Surgery
IAP: Intra-Abdominal Pressure
IBW: Ideal Body Weight
LSRS: Leiden Surgical Rating Scale
PIP: Peak Inspiratory Pressure
Pplat: Plateau Pressure
PEEP: Positive End-Expiratory Pressure
TV: Tidal Volume
Raw: Airway Resistance
ΔP: Driving Pressure (ΔP = Pplat - PEEP)
Cdyn: Dynamic Lung Compliance
ASA: American Society of Anesthesiologists
MAP: Mean Arterial Pressure
HR: Heart Rate
SPO_2_: Oxygen Saturation
BIS: Bispectral Index
NRS: Numeric Rating Scale
TOF: Train Of Four
PTC: Post Tetanic Count
