# Supplementary figures and images for: Deep Neuromuscular Blockade in Laparoscopic One-Anastomosis Gastric Bypass (OAGB): A Randomized Controlled Trial
Source: Obes Surg. 2026 Jun 16;36(8):4186–97. doi: 10.1007/s11695-026-08799-8 (PMC13429543; doi:10.1007/s11695-026-08799-8)

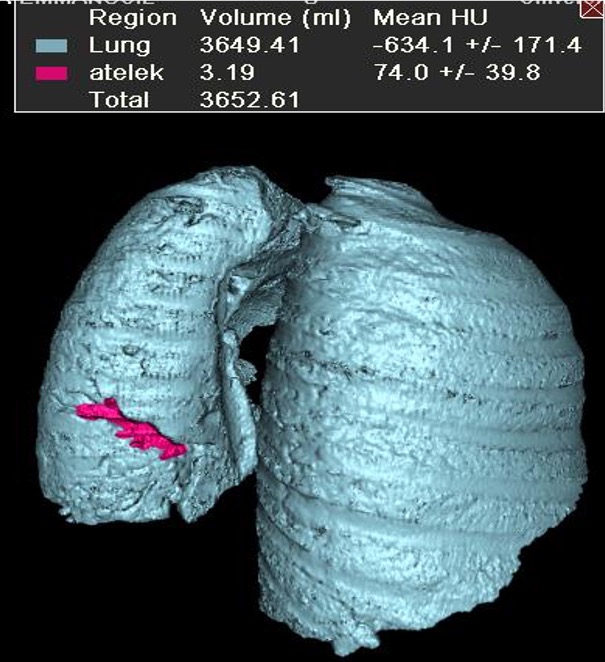

Supplement: Supplementary file 1 — Supplementary Material 1 [file 11695_2026_8799_MOESM1_ESM.jpg]

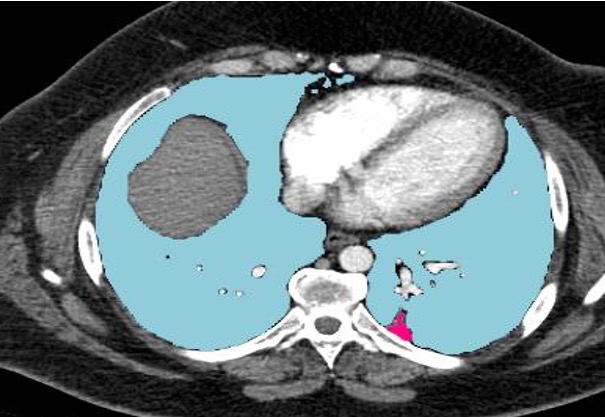

Supplement: Supplementary file 2 — Supplementary Material 2 [file 11695_2026_8799_MOESM2_ESM.jpg]
