# Supplementary material for: Deep Neuromuscular Blockade in Laparoscopic One-Anastomosis Gastric Bypass (OAGB): A Randomized Controlled Trial
Source: Obes Surg. 2026 Jun 16;36(8):4186–97. doi: 10.1007/s11695-026-08799-8 (PMC13429543; doi:10.1007/s11695-026-08799-8)
Supplement: Supplementary file 3 — Supplementary Material 3 [file 11695_2026_8799_MOESM3_ESM.docx]

**Supplementary material**

| **Supplementary Table 1: Ventilation parameters during operation** | | |
| --- | --- | --- |
| **Parameter** | **Change during operation (p-value)** | **Interaction with neuromuscular blockade depth (p-value)** |
| PIP | **<0.001** | 0.957 |
| Plat | **<0.001** | 0.874 |
| Map | **<0.001** | 0.987 |
| DP | **<0.001** | 0.825 |
| Raw | **<0.001** | 0.989 |
| Cd | **<0.001** | 0.966 |

PIP: peak inspiratory pressure, Pplat: plateau pressure, Map: mean airway pressure, DP: driving pressure, Raw: airway resistance, Dd: dynamic compliance

| **Supplementary Table 2: Intraoperative Parameters (Surgical Field, Duration, and Vital Signs)** | | | |
| --- | --- | --- | --- |
| **Parameter** | **Moderate neuromuscular blockade** | **Deep Neuromuscular blockade** | **P-value** |
| Average Leiden Surgical Rating Scale score | Mean (SD): 3.8 (0.6)  Median (IQR): 3.8 (0.5) | Mean (SD): 4.5 (0.5)  Median (IQR): 4.7 (0.6) | **<0.001** |
| Operating time (min) | Mean (SD): 71.8 (17.5)  Median (IQR): 65 (29) | Mean (SD): 60.8 (21.8)  Median (IQR): 55 (20) | **0.007** |
| Average SpO_2_ (%) | Mean (SD): 97.9 (1.5)  Median (IQR): 97.8 (2.2) | Mean (SD): 97.9 (1.7)  Median (IQR): 98.2 (1.7) | 0.705 |
| Average heart rate (bpm) | Mean (SD): 72 (10)  Median (IQR): 73 (15) | Mean (SD): 77 (11)  Median (IQR): 78 (18) | 0.095 |
| Average SBP (mmHg) | Mean (SD): 125 (16)  Median (IQR): 126 (19) | Mean (SD): 125 (14)  Median (IQR): 126 (23) | 0.907 |
| Average DBP (mmHg) | Mean (SD): 73 (11)  Median (IQR): 73 (10) | Mean (SD): 71 (8)  Median (IQR): 70 (13) | 0.35 |
| Average MAP (mmHg) | Mean (SD): 90 (12)  Median (IQR): 92 (12) | Mean (SD): 89 (9)  Median (IQR): 87 (17) | 0.539 |

MNB: moderate neuromuscular blockade, DNB: deep neuromuscular blockade, SBP: systolic blood pressure, DBP: diastolic blood pressure, MAP: mean arterial pressure

| **Supplementary Table 3: Multivariable linear regression analysis for average Leiden Surgical Rating Scale score** | | | |
| --- | --- | --- | --- |
| Parameter | Beta Coefficient | 95% CI | P-value |
| Neuromuscular blockade grade  (Reference: Moderate)  Deep | 0.737 | 0.443 – 1.031 | **<0.001** |
| Gender  (Reference: female)  Male | 0.302 | 0.009 – 0.595 | **0.043** |
| Age (years) | 0.005 | -0.009 – 0.018 | 0.482 |
| BMI | -0.016 | -0.036 – 0.004 | 0.117 |

BMI: body mass index

| **Supplementary Table 4: Multivariable linear regression analysis for operating time (min)** | | | |
| --- | --- | --- | --- |
| Parameter | Beta Coefficient | 95% CI | P-value |
| Neuromuscular blockade grade  (Reference: Moderate)  Deep | -11.529 | -22.737 – -0.321 | **0.044** |
| Gender  (Reference: female)  Male | -0.253 | -11.412 – 10.906 | 0.964 |
| Age (years) | 0.267 | -0.252 – 0.786 | 0.306 |
| BMI | 0.335 | -0.431 – 1.101 | 0.384 |

BMI: body mass index

| **Supplementary Table 5: Postoperative Outcomes Including Vital Signs, Pain Assessment, Analgesic Use, Return of Flatus, and Length of Hospital Stay.** | | | |
| --- | --- | --- | --- |
| **Parameter** | **Moderate neuromuscular blockade** | **Deep Neuromuscular blockade** | **P-value** |
| Average SpO_2_ (%) | Mean (SD): 97.9 (1.5)  Median (IQR): 97.8 (2.4) | Mean (SD): 98.3 (1.7)  Median (IQR): 98.8 (1.5) | 0.166 |
| Average heart rate (bpm) | Mean (SD): 75 (9)  Median (IQR): 73 (14) | Mean (SD): 80 (29)  Median (IQR): 77 (16) | 0.485 |
| Average SBP (mmHg) | Mean (SD): 146 (17)  Median (IQR): 149 (21) | Mean (SD): 143 (13)  Median (IQR): 141 (20) | 0.522 |
| Average DBP (mmHg) | Mean (SD): 77 (7)  Median (IQR): 78 (10) | Mean (SD): 75 (8)  Median (IQR): 76 (11) | 0.324 |
| Average MAP (mmHg) | Mean (SD): 100 (9)  Median (IQR): 102 (10) | Mean (SD): 98 (8)  Median (IQR): 97 (8.5) | 0.334 |
| Average pain score (1^st^ hour) | Mean (SD): 4.5 (1.5)  Median (IQR): 4.6 (1.9) | Mean (SD): 3.7 (1.4)  Median (IQR): 3.5 (2) | **0.032** |
| Pain score at 8pm (same day) | Mean (SD): 3.4 (1.3)  Median (IQR): 3 (1) | Mean (SD): 2.3 (1.1)  Median (IQR): 2 (2) | **0.002** |
| Pain score at 2pm (next day) | Mean (SD): 1.9 (1.1)  Median (IQR): 2 (2) | Mean (SD): 1.3 (0.5)  Median (IQR): 1 (0) | **0.009** |
| Total parecoxib dose (mg) | Mean (SD): 92.3 (24.7)  Median (IQR): 80 (40) | Mean (SD): 84.4 (30)  Median (IQR): 80 (0) | 0.129 |
| Total pethidine dose (mg) | Mean (SD): 101.9 (53.8)  Median (IQR): 100 (100) | Mean (SD): 75.9 (37.6)  Median (IQR): 100 (50) | 0.053 |
| Days to flatus | Mean (SD): 1.5 (0.6)  Median (IQR): 1.5 (1) | Mean (SD): 1.3 (0.8)  Median (IQR): 1 (0) | **0.044** |
| Hospital stay duration (days) | Mean (SD): 3.1 (0.3)  Median (IQR): 3 (0) | Mean (SD): 3.3 (1.2)  Median (IQR): 3 (0) | 0.879 |

MNB: moderate neuromuscular blockade, DNB: deep neuromuscular blockade, SBP: systolic blood pressure, DBP: diastolic blood pressure, MAP: mean arterial pressure

| **Supplementary Table 6: Multivariable linear regression analysis for average pain score during the first postoperative hour** | | | |
| --- | --- | --- | --- |
| **Parameter** | **Beta Coefficient** | **95% CI** | **P-value** |
| Neuromuscular blockade grade  (Reference: Moderate)  Deep | -1.076 | -2.009 – -0.144 | **0.025** |
| Gender  (Reference: female)  Male | -0.551 | -1.48 – 0.378 | 0.238 |
| Age (years) | -0.006 | -0.05 – 0.038 | 0.77 |
| BMI | -0.008 | -0.07 – 0.054 | 0.798 |
| Operating time (min) | -0.013 | -0.036 – 0.011 | 0.296 |
| Fentanyl (mg) | 0.001 | -0.004 – 0.006 | 0.612 |
| Remifentanil (mg) | 0.0002 | -0.001 – 0.001 | 0.695 |

| **Supplementary Table 7: Multivariable linear regression analysis for pain score at 8pm on the same day** | | | |
| --- | --- | --- | --- |
| **Parameter** | **Beta Coefficient** | **95% CI** | **P-value** |
| Neuromuscular blockade grade  (Reference: Moderate)  Deep | -1.06 | -1.754 – -0.365 | **0.004** |
| Gender  (Reference: female)  Male | -0.317 | -1.009 – 0.375 | 0.361 |
| Age (years) | -0.001 | -0.034 – 0.031 | 0.933 |
| BMI | -0.07 | -0.116 – -0.024 | **0.004** |
| Operating time (min) | -0.008 | -0.026 – 0.01 | 0.355 |
| Fentanyl (mg) | 0.002 | -0.001 – 0.006 | 0.236 |
| Remifentanil (mg) | 0.00009 | -0.001 – 0.001 | 0.832 |

| **Supplementary Table 8: Multivariable linear regression analysis for pain score at 2pm on the following day** | | | |
| --- | --- | --- | --- |
| **Parameter** | **Beta Coefficient** | **95% CI** | **P-value** |
| Neuromuscular blockade grade  (Reference: Moderate)  Deep | -0.767 | -1.268 – -0.266 | **0.004** |
| Gender  (Reference: female)  Male | -0.561 | -1.06 – -0.062 | **0.028** |
| Age (years) | 0.004 | -0.019 – 0.028 | 0.711 |
| BMI | -0.032 | -0.066 – 0.001 | 0.057 |
| Operating time (min) | -0.009 | -0.021 – 0.004 | 0.187 |
| Fentanyl (mg) | -0.00002 | -0.003 – 0.003 | 0.986 |
| Remifentanil (mg) | 0.0003 | -0.0004 – 0.001 | 0.391 |

| **Supplementary Table 9: Multivariable linear regression analysis for atelectasis volume (ml)** | | | |
| --- | --- | --- | --- |
| **Parameter** | **Beta Coefficient** | **95% CI** | **P-value** |
| Neuromuscular blockade grade  (Reference: Moderate)  Deep | -3.643 | -25.948 – 18.662 | 0.743 |
| Gender  (Reference: female)  Male | 24.542 | 2.991 – 46.093 | **0.027** |
| Age (years) | 0.611 | -0.396 – 1.619 | 0.228 |
| BMI | -0.414 | -1.898 – 1.07 | 0.577 |
| Smoking  (Reference: no)  Yes | 5.842 | -15.825 – 27.509 | 0.589 |
| Operating time (min) | -0.44 | -0.981 – 0.102 | 0.109 |

| **Supplementary Table 10: Multivariable linear regression analysis for atelectasis percentage (%)** | | | |
| --- | --- | --- | --- |
| **Parameter** | **Beta Coefficient** | **95% CI** | **P-value** |
| Neuromuscular blockade grade  (Reference: Moderate)  Deep | -0.489 | -1.539 – 0.561 | 0.353 |
| Gender  (Reference: female)  Male | 0.193 | -0.821 – 1.208 | 0.703 |
| Age (years) | 0.007 | -0.041 – 0.054 | 0.773 |
| BMI | -0.008 | -0.078 – 0.062 | 0.81 |
| Smoking  (Reference: no)  Yes | 0.122 | -0.898 – 1.142 | 0.81 |
| Operating time (min) | -0.015 | -0.041 – 0.01 | 0.237 |
